# Supplementary figures and images for: MTH1 deficiency selectively increases non-cytotoxic oxidative DNA damage in lung cancer cells: more bad news than good?
Source: BMC Cancer. 2018 Apr 16;18:423. doi: 10.1186/s12885-018-4332-7 (PMC5903006; doi:10.1186/s12885-018-4332-7)

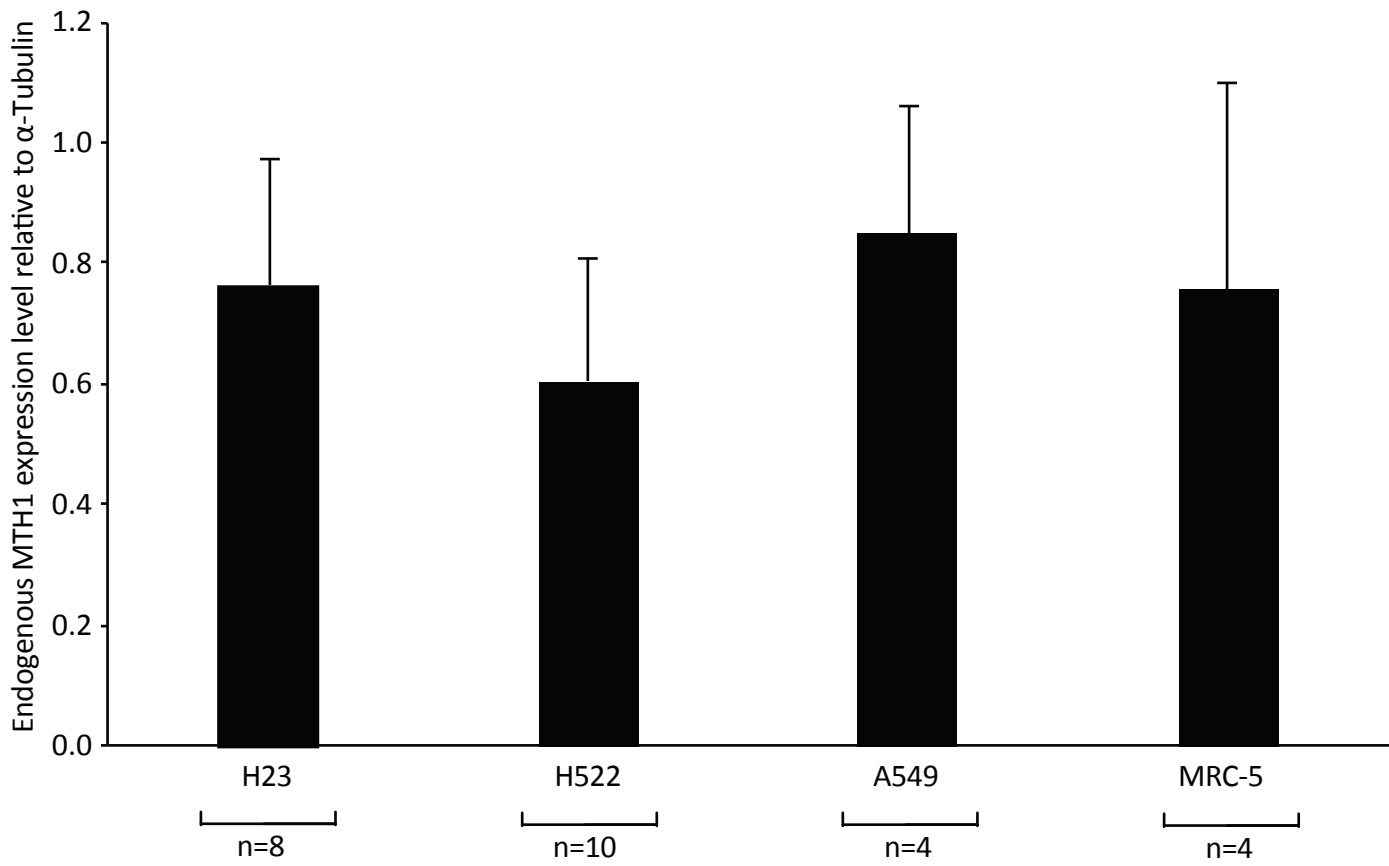

Supplement: Supplementary file 1 — Endogenous MTH1 levels are similar in H23, H522, A549 and MRC-5 cell lines. MTH1 band intensities in no siRNA samples were normalized to corresponding α-Tubulin loading control bands (see Fig. 1 for representative Western blot images). Mean values and SD were calculated from the normalised values of independent experiments. Numbers of independent experiments (n) are indicated. Error bars represent SD. (PDF 221 kb) [file 12885_2018_4332_MOESM1_ESM.pdf]

## H23

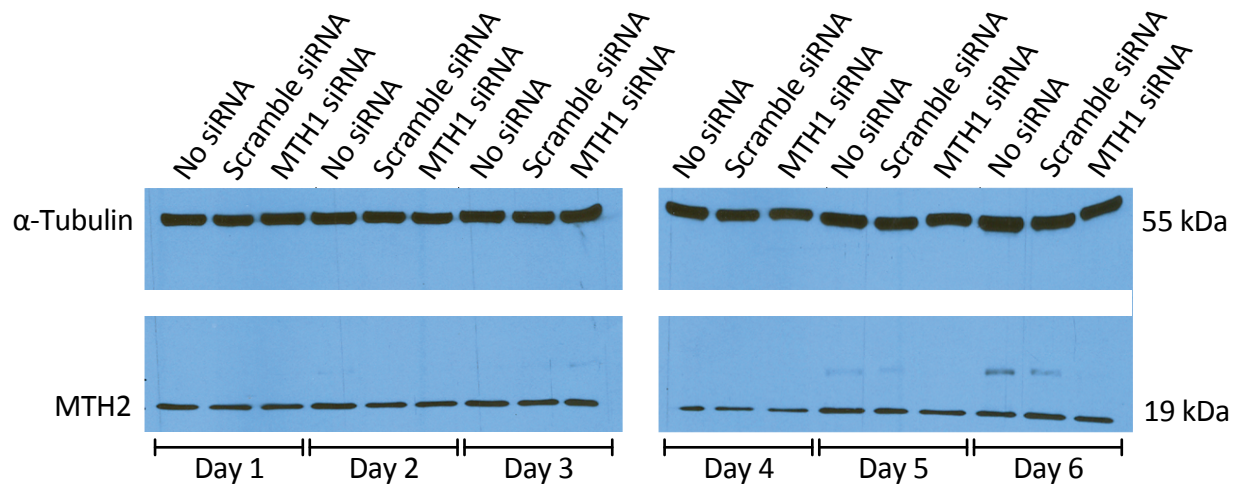

Supplement: Supplementary file 2 — MTH2 levels are stable following MTH1 siRNA knockdown. Western blots from a single confirmation experiment performed to determine MTH2 protein levels in cell cultures grown in media without transfection reagent (no siRNA), or following transfection with MTH1 siRNA or scramble siRNA (n = 1). (PDF 1128 kb) [file 12885_2018_4332_MOESM2_ESM.pdf]

(i)

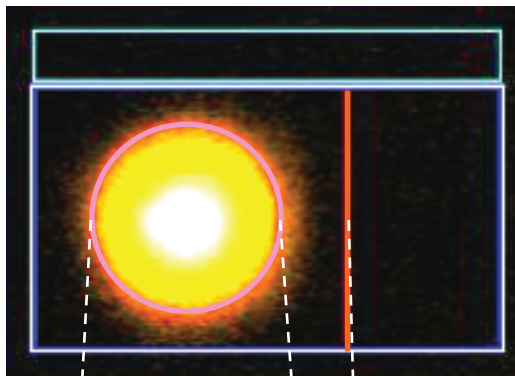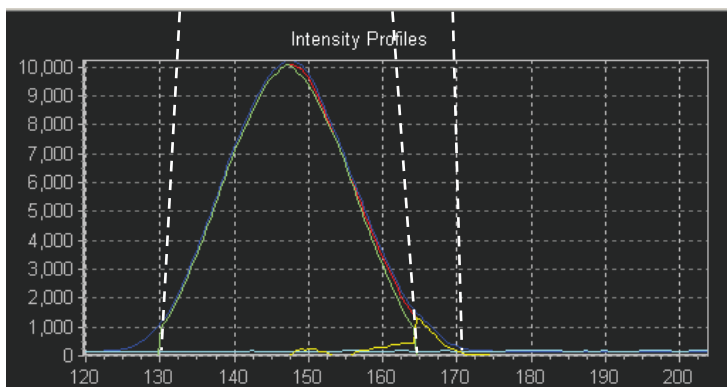

(ii)

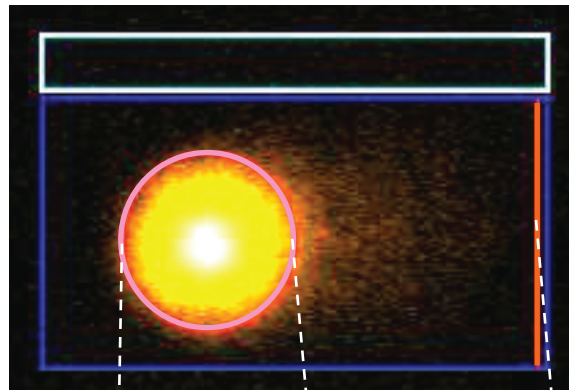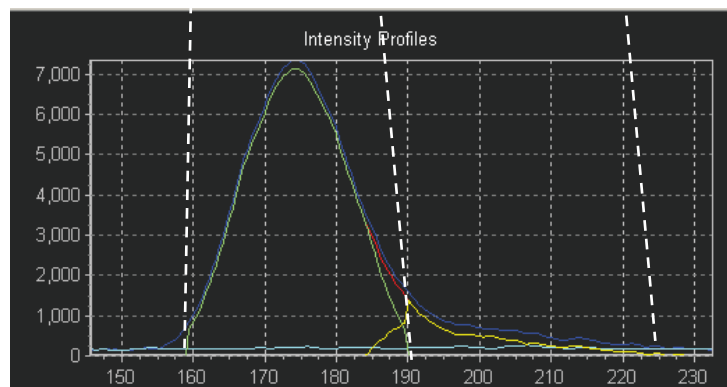

Supplement: Supplementary file 3 — Representative images of “Comets” and the corresponding intensity profiles, showing (i) ~ 5% Tail DNA damage, typical of the NSCLC cells treated with no siRNA or scramble siRNA, and analysed by regular Fpg-modified alkaline comet assay (0.8 U Fpg/gel); and (ii) comets showing ~ 10% tail DNA, typical of the NSCLC cells treated with MTH1 siRNA. Superimposed on the Comet images are the image analysis software (Komet 5.5, Andor Technology) determined boundaries demarcating the ‘Comet head’ (pink circle) and ‘tail extent’ (vertical orange line) (Barber RC, Hickenbotham P, Hatch T, Kelly D, Topchiy N, Almeida GM, et al. Radiation-induced transgenerational alterations in genome stability and DNA damage. Oncogene. 2006;25(56):7336–7342). % tail DNA = 100 - % head DNA; % head DNA = (integrated optical head intensity / (integrated optical head intensity + integrated optical tail intensity)) × 100. (PDF 1431 kb) [file 12885_2018_4332_MOESM3_ESM.pdf]

**a**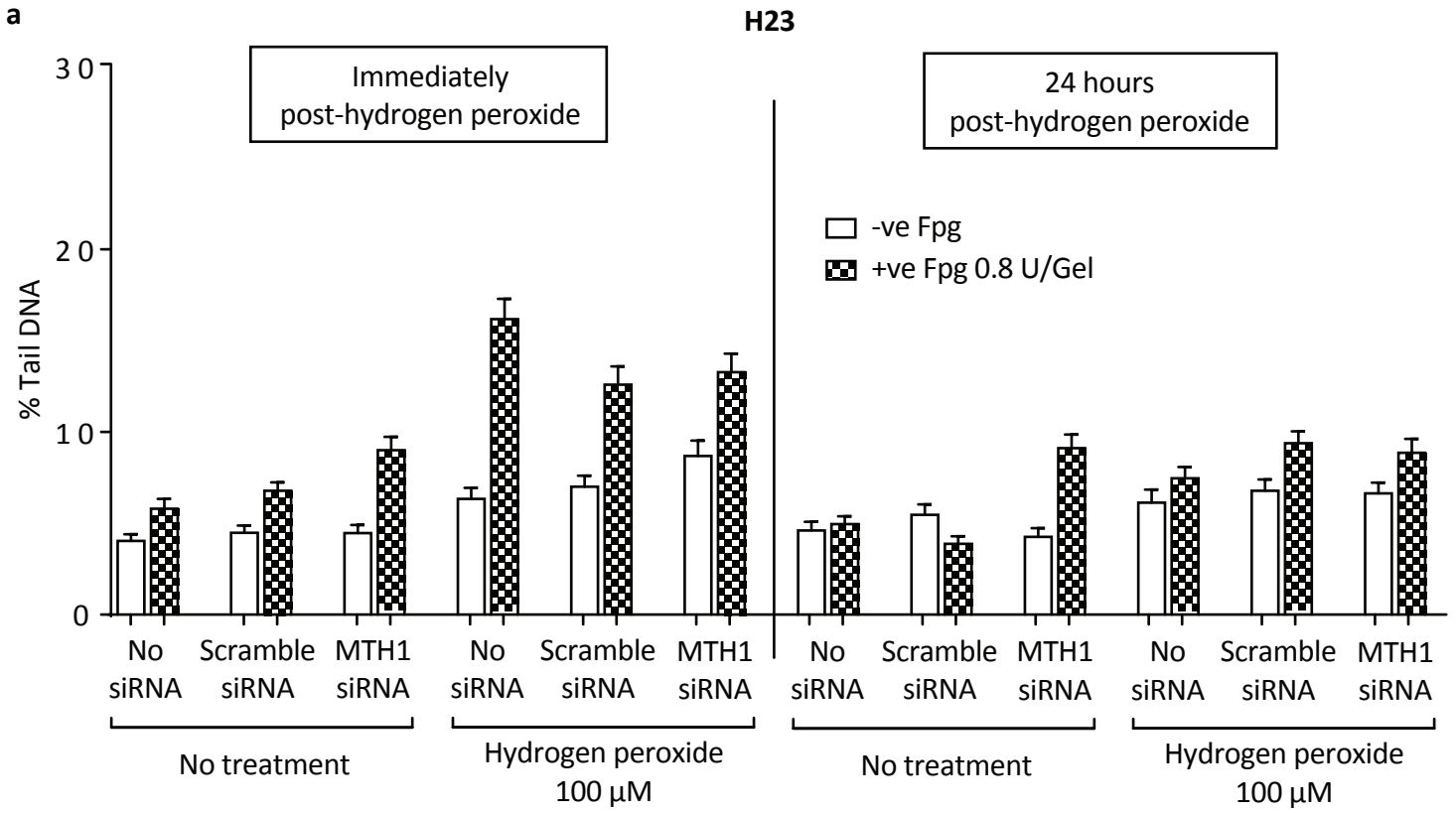**b**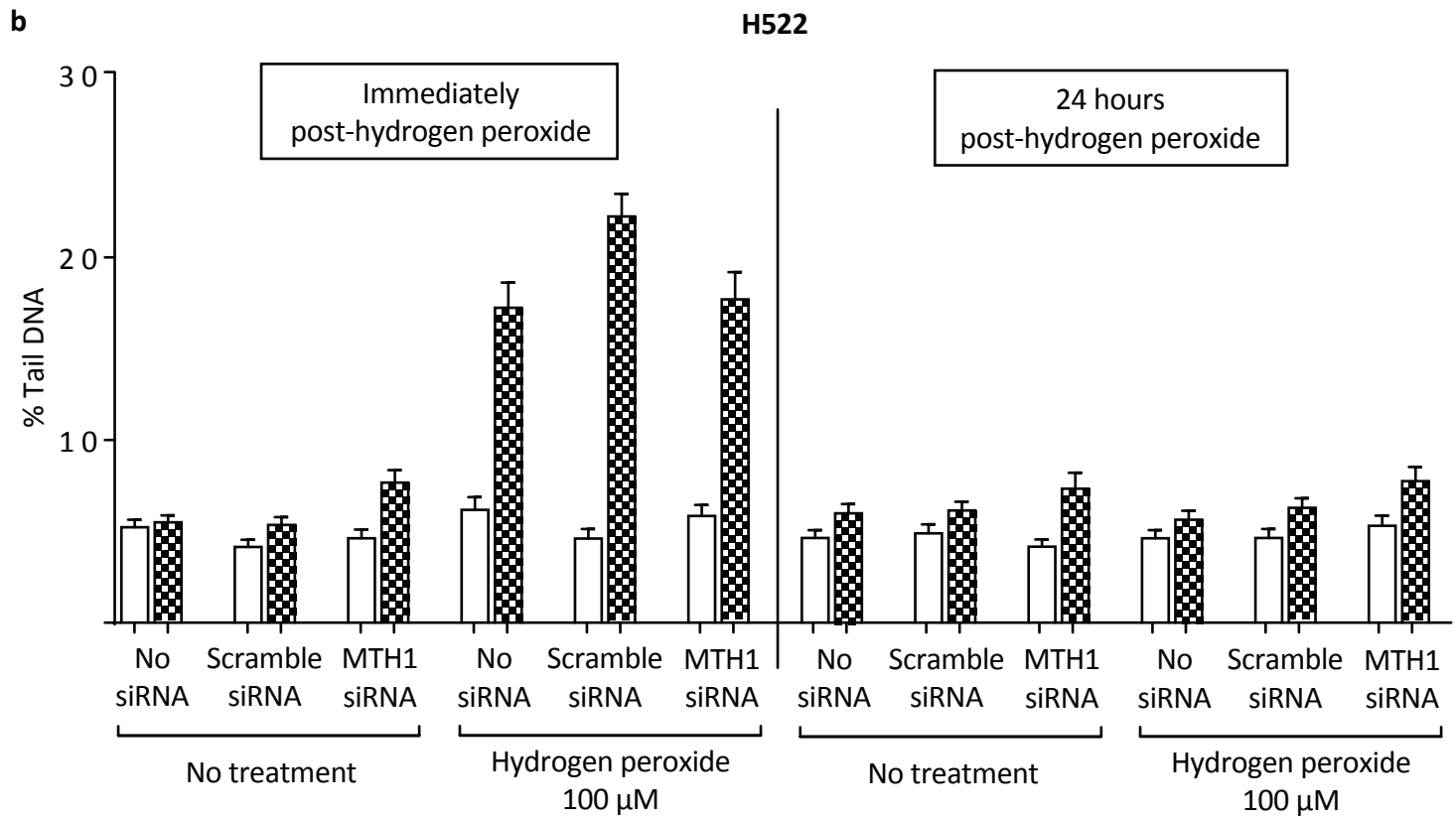

Supplement: Supplementary file 4 — MTH1 deficiency does not alter levels of oxidatively-modified DNA following hydrogen peroxide treatment. Fpg-modified alkaline comet assay to determine DNA damage levels in individual cells grown in media without transfection reagent (no siRNA), or 4 days after transfection with MTH1 siRNA or scramble siRNA. After 30 min hydrogen peroxide treatment at 37 °C, samples were collected either immediately or allowed to recover in fresh media. Means were calculated from 100 individual comets from a single experiment. Error bars represent SEM of comet values. a H23. b H522. (PDF 328 kb) [file 12885_2018_4332_MOESM4_ESM.pdf]
