# Supplementary material for: MTH1 deficiency selectively increases non-cytotoxic oxidative DNA damage in lung cancer cells: more bad news than good?
Source: BMC Cancer. 2018 Apr 16;18:423. doi: 10.1186/s12885-018-4332-7 (PMC5903006; doi:10.1186/s12885-018-4332-7)
Supplement: Supplementary file 5 — MTH1 knockdown with another siRNA similarly does not induce apoptosis in H23 cells. a Western blots to determine MTH1 protein levels in H23 cell cultures grown in media without transfection reagent (no siRNA), or following transfection with MTH1 siRNA (ThermoFisher Scientific, S194633, oligonucleotide 5′- > 3′ sequences were sense UUAACUGGAUGGAAGGGAAtt and antisense AUCCAGUUAAUUCCAGAUGaa) or scramble siRNA. Representative day 4 blot shown. Day 4 MTH1 band intensities were normalized to corresponding α-Tubulin loading control bands, and then siRNA samples were normalised to corresponding no siRNA bands. Numbers of independent experiments (n) are indicated. Mean values were calculated from the normalised values of the independent experiments. Error bars represent SD. Asterisks represent a significant difference between MTH1 siRNA and corresponding no siRNA normalised values (**P < 0.01, *P < 0.05). b Apoptosis assay to determine cell viability of H23 cells cultured for 4 days in media without transfection reagent (no siRNA), or following transfection with MTH1 siRNA (S194633) or scramble siRNA. Harvested cells were dual stained with annexin V-FITC/PI and assessed by flow cytometry to detect both early and late apoptosis. 2 days after transfection, 0.01 μM gemcitabine (Gem) or 5 μM cisplatin (Cis) were added to the appropriate cultures for the remaining 48 h. Positive control of 48 h VP-16 treatment (+ve) also included (n = 1). (PDF 436 kb) [file 12885_2018_4332_MOESM5_ESM.pdf]

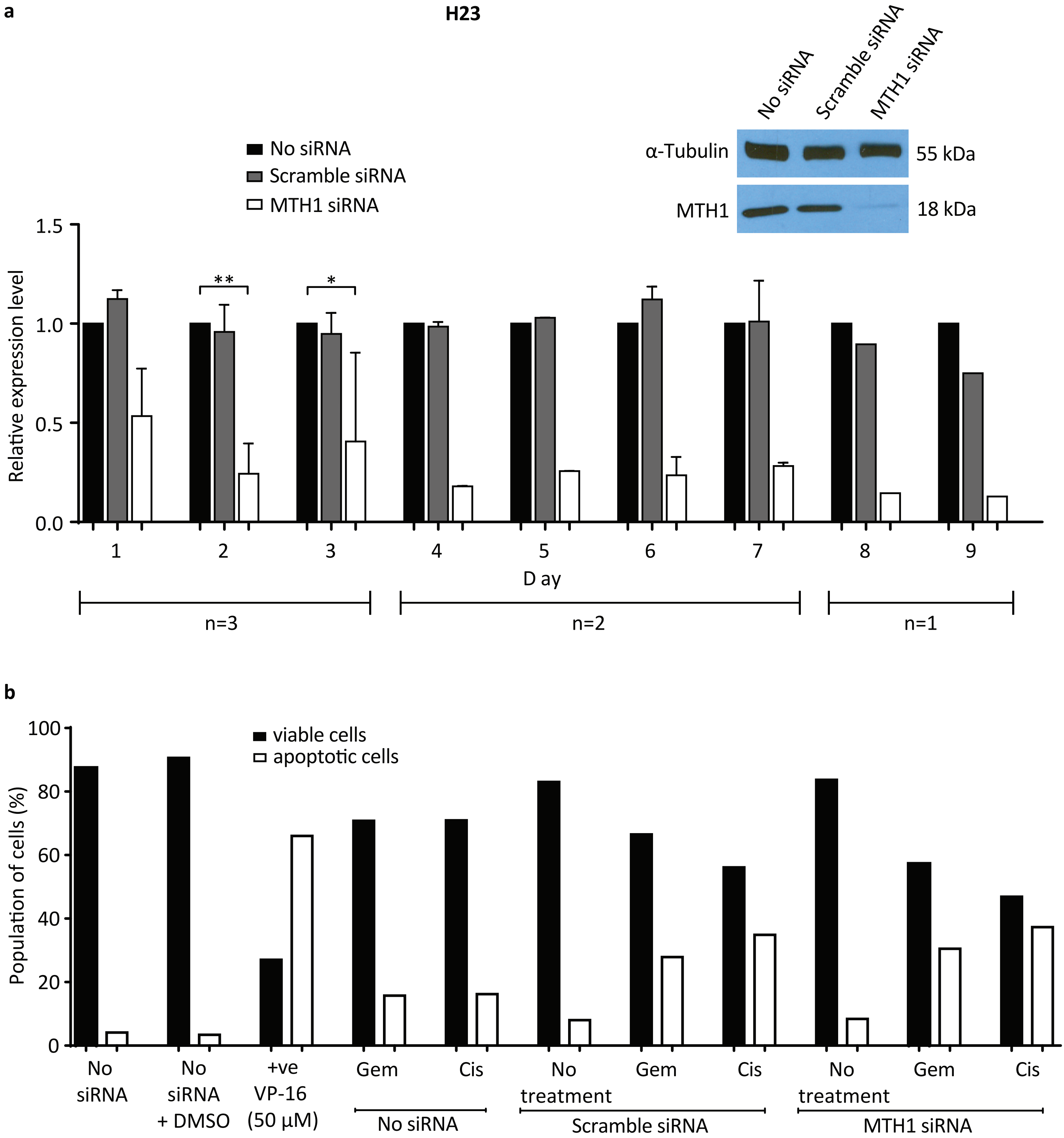
a
H23
No siRNA
Scramble siRNA
MTH1 siRNA
α-Tubulin
55 kDa
MTH1
18 kDa
No siRNA
Scramble siRNA
MTH1 siRNA
Relative expression level
1.5
1.0
0.5
0.0
**
*
1
2
3
4
5
6
7
8
9
D ay
n=3
n=2
n=1
b
viable cells
apoptotic cells
Population of cells (%)
100
80
60
40
20
0
No siRNA
No siRNA + DMSO
+ve VP-16 (50 µM)
Gem
Cis
No siRNA
No treatment
Gem
Cis
Scramble siRNA
No treatment
Gem
Cis
MTH1 siRNA
