# Supplementary material for: MTH1 deficiency selectively increases non-cytotoxic oxidative DNA damage in lung cancer cells: more bad news than good?
Source: BMC Cancer. 2018 Apr 16;18:423. doi: 10.1186/s12885-018-4332-7 (PMC5903006; doi:10.1186/s12885-018-4332-7)
Supplement: Supplementary file 6 — Apoptotic dose responses of H23 and H522 cell lines to various genotoxic agents. Apoptosis assay to determine cell viability following 48 h treatment with different agents. In addition, cells were exposed to VP-16 for positive controls, and DMSO (0.5–2% v/v) and untreated negative control samples were also included. Harvested cells were dual stained with annexin V-FITC/PI and assessed by flow cytometry to detect both early and late apoptosis. a Hydrogen peroxide treatment of H23 and H522 cells. b Gemcitabine treatment of H23 and H522 cells. c Cisplatin treatment of H23 and H522 cells. Percentage values from independent experiments were used to calculate final mean values and SD. Error bars represent SD. All experiments were repeated more than 3 times. Asterisks indicate a significant difference relative to corresponding DMSO controls (****P < 0.0001, ***P < 0.001, **P < 0.01, and *P < 0.05). (PDF 423 kb) [file 12885_2018_4332_MOESM6_ESM.pdf]

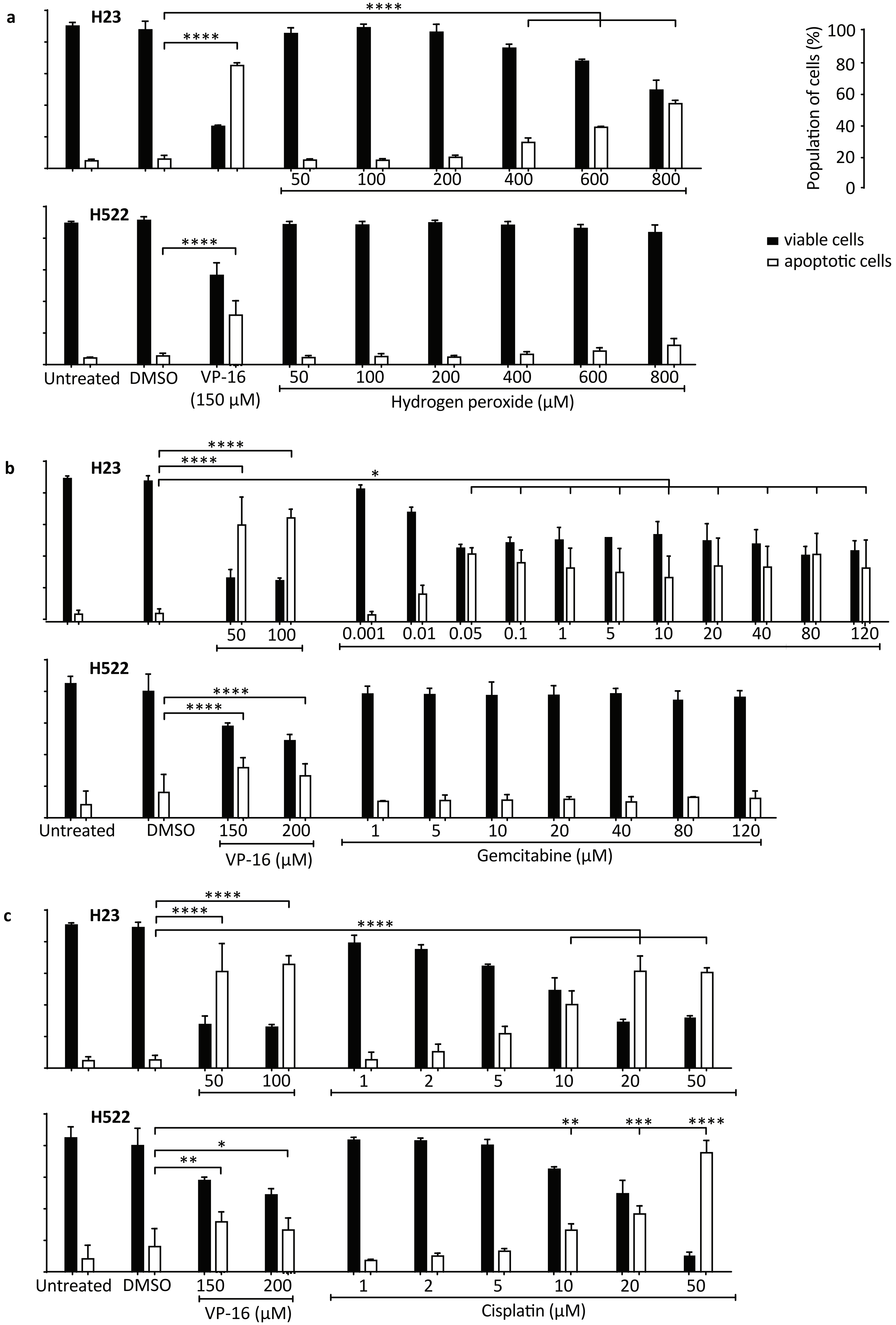
a
H23
****
****
50
100
200
400
600
800
Population of cells (%)
100
80
60
40
20
0
H522
****
viable cells
apoptotic cells
Untreated
DMSO
VP-16
(150 µM)
50
100
200
400
600
800
Hydrogen peroxide (µM)
b
H23
****
****
*
50
100
0.001
0.01
0.05
0.1
1
5
10
20
40
80
120
H522
****
****
Untreated
DMSO
150
200
VP-16 (µM)
1
5
10
20
40
80
120
Gemcitabine (µM)
c
H23
****
****
****
50
100
1
2
5
10
20
50
H522
**
***
****
*
**
Untreated
DMSO
150
200
VP-16 (µM)
1
2
5
10
20
50
Cisplatin (µM)
